# Pangenome-spanning epistasis and coselection analysis via de Bruijn graphs

**DOI:** 10.1101/gr.278485.123

**Published:** 2024-07

**Authors:** Juri Kuronen, Samuel T. Horsfield, Anna K. Pöntinen, Sudaraka Mallawaarachchi, Sergio Arredondo-Alonso, Harry Thorpe, Rebecca A. Gladstone, Rob J.L. Willems, Stephen D. Bentley, Nicholas J. Croucher, Johan Pensar, John A. Lees, Gerry Tonkin-Hill, Jukka Corander

**Affiliations:** 1 Department of Biostatistics, University of Oslo, 0372 Blindern, Norway;; 2 MRC Centre for Global Infectious Disease Analysis, Department of Infectious Disease Epidemiology, Imperial College London, London W12 0BZ, United Kingdom;; 3 European Molecular Biology Laboratory, European Bioinformatics Institute, Wellcome Genome Campus, Hinxton CB10 1SD, United Kingdom;; 4 Norwegian National Advisory Unit on Detection of Antimicrobial Resistance, Department of Microbiology and Infection Control, University Hospital of North Norway, 9019 Tromsø, Norway;; 5 Peter MacCallum Cancer Centre, Melbourne, Victoria 3052, Australia;; 6 Sir Peter MacCallum Department of Oncology, University of Melbourne, Melbourne, Victoria 3052, Australia;; 7 Department of Medical Microbiology, University Medical Center Utrecht, 3584 CX Utrecht, Netherlands;; 8 Parasites and Microbes, Wellcome Sanger Institute, Cambridge CB10 1RQ, United Kingdom;; 9 Department of Mathematics, University of Oslo, 0372 Blindern, Norway;; 10 Department of Microbiology and Immunology, The University of Melbourne, at the Peter Doherty Institute for Infection and Immunity, Melbourne, Victoria 3052, Australia;; 11 Helsinki Institute of Information Technology, Department of Mathematics and Statistics, University of Helsinki, 00014 Helsinki, Finland

## Abstract

Studies of bacterial adaptation and evolution are hampered by the difficulty of measuring traits such as virulence, drug resistance, and transmissibility in large populations. In contrast, it is now feasible to obtain high-quality complete assemblies of many bacterial genomes thanks to scalable high-accuracy long-read sequencing technologies. To exploit this opportunity, we introduce a phenotype- and alignment-free method for discovering coselected and epistatically interacting genomic variation from genome assemblies covering both core and accessory parts of genomes. Our approach uses a compact colored de Bruijn graph to approximate the intragenome distances between pairs of loci for a collection of bacterial genomes to account for the impacts of linkage disequilibrium (LD). We demonstrate the versatility of our approach to efficiently identify associations between loci linked with drug resistance and adaptation to the hospital niche in the major human bacterial pathogens *Streptococcus pneumoniae* and *Enterococcus faecalis*.

Epistatic interactions between polymorphisms in DNA are recognized as important drivers of evolution in numerous organisms. It was recently established that even weak to moderate coselective and epistatic effects may manifest themselves in bacteria, in contrast with eukaryotes, in which generally only stronger effects bring sufficient selective advantages (Arnold et al. 2018). The rapidly increasing amount of whole-genome data for many named species of bacteria has opened up a possibility to identify such effects on a pangenomic level. Recent approaches to genome-scale analysis of covariation at single-nucleotide resolution, termed as the genome-wide epistasis and coselection study (GWES), have demonstrated ample potential to uncover drivers of adaptation, virulence, survival, and antimicrobial resistance from densely sampled populations of major pathogens (Skwark et al. 2017; Pensar et al. 2019; Top et al. 2020; Chewapreecha et al. 2022).

The GWES approach can be considered as a phenotype-free biological hypothesis generator that works in a complementary manner compared with genome-wide association study (GWAS), which also aims to generate hypotheses of causal drivers. The aim of GWES studies is to identify potential sites that are coevolving under a common selective pressure and may be, although not necessarily, involved in epistatic interactions. GWAS works by correlating genomic variation with measured phenotypic variation and has been widely applied to the study of bacterial traits, most notably antibiotic resistance (Earle et al. 2016; Lees et al. 2018, 2020). The availability of standardized and accurate bacterial phenotyping is often limited, focusing primarily on specific phenotypes like antibiotic resistance, as more opaque phenotypes like transmissibility, hospital adaptation, and virulence are harder to quantify. GWES holds considerable promise to address these difficulties by directly measuring coevolutionary signals, driving molecular discoveries. Existing GWES approaches have predominantly relied upon multiple sequence alignments (MSAs) produced by mapping reads to a single-reference genome. This neglects signals found in accessory elements that are absent from the reference genome, including short indels and variation found in accessory genes. Additionally, missing data in alignments can be difficult to handle correctly, and genotyping quality can vary between samples owing to the choice of thresholds used for filtering. To overcome these shortcomings and expand the potential of GWES, we developed an alignment-free approach using colored de Bruijn graphs to distinguish between short- and long-distance linkage disequilibrium (LD) in bacterial genomes.

Use of de Bruijn graphs in genome assembly, reference genome representation, and variant calling has become commonplace in population genomics, in particular for bacteria (Bankevich et al. 2012; Holley and Melsted 2020; Colquhoun et al. 2021). A colored and compacted de Bruijn graph (cdBG) offers a powerful representation of the variation present across a collection of genome assemblies that can be utilized for a wide range of applications, including GWAS (Jaillard et al. 2018; Lees et al. 2020) and genotyping (Iqbal et al. 2012). Within a colored cdBG, variable length sequences, or “unitigs,” represent loci within each genome, with the color indicating the genome to which the unitig belongs. As a measure of genomic variation within a population, unitigs are advantageous as they can efficiently tag a range of different types of changes, including mutations, indels, and variation in gene content, all of which represent important processes in evolution and adaptation of bacteria. Nevertheless, obtaining a colored cdBG for a large set of input genome assemblies is an intensive computational problem, which has only recently become accessible owing to algorithmic advances (Holley and Melsted 2020; Khan and Patro 2021; Cracco and Tomescu 2023). One recent colored de Bruijn graph construction algorithm, Cuttlefish, provides an optimal starting point for a pangenomic GWES that does not rely on a single-reference genome-based representation of variation (Khan and Patro 2021). Using this data structure, we have developed an efficient algorithm, PAN-GWES, that computes approximate distances between loci within a pangenome and estimates the signal of epistasis or coselection between loci using mutual information (MI). Applications to large *Streptococcus pneumoniae* and *Enterococcus faecalis* genomic data sets revealed not only previously identified but also new indications of coselection between genomic loci, linked to antibiotic resistance and adaptation to hospital environments.

## Results

### Overview

**Figure 1.**
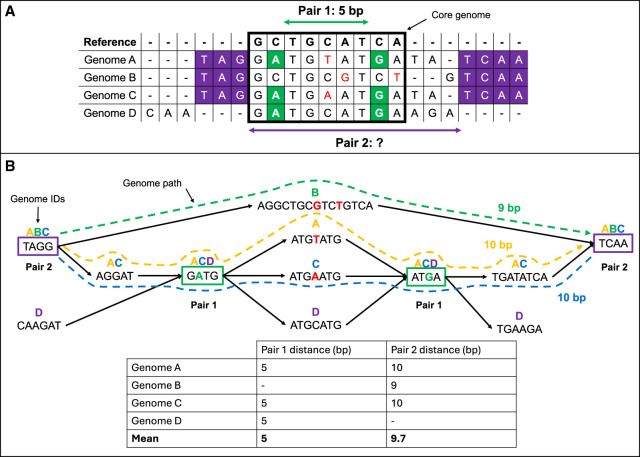
Comparison of MSA- and DBG-based GWES. (*A*) MSA-based GWES uses distances calculated from alignment of genomes to a reference. Only variants found within blocks of sequence shared with the reference, for example, within the core genome, can be detected and have distances calculated between them, exemplified by pair 1. Pair 2, which is variants found within the accessory genome, do not align to the reference, meaning the distance between them cannot be calculated and so is ignored from the mutual information (MI) calculation. (*B*) Using a DBG, distances can be calculated for pair 2 by traversing the graph for each genome containing this pair of variant, and then by calculating the mean distance between them. The DBG can also be used to calculate the distance for pair 1 using the same approach. Therefore, variant pairs in both the core and accessory genome can be identified.

**Figure 2.**
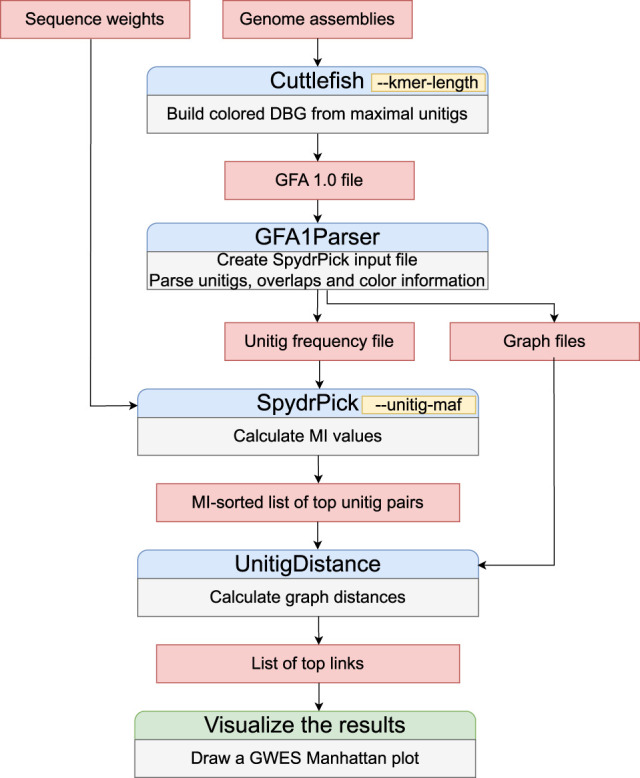
Overview of the PAN-GWES pipeline with the main user-definable parameters highlighted. Assemblies are the only required input, enabling the PAN-GWES to generate phenotype-free biological hypotheses in a complementary manner to traditional GWAS methods.

Our PAN-GWES algorithm starts by constructing a colored cdBG of all genome assemblies, as depicted in Figure 1. Figure 2 provides a comprehensive overview of the various stages and adjustable parameters within the algorithm. The colored cdBG is initially built using the computationally efficient Cuttlefish algorithm with a given *k*-mer length. LD between pairs of variants, represented as nodes or unitigs in the graph, is measured using MI, with additional weighting to account for population structure as described previously (Pensar et al. 2019). The MI-based method is specifically designed to detect long-distance pairs that show an elevated association with respect to a background distribution and, thus, relies on a measure of genomic distance to filter out short-distance pairs.

Without a MSA based on a single-reference genome, it is not straightforward to define a unique distance between variants observed in a population of genomes (Fig. 1A). To approximate the distance between a pair of unitigs, we compute the distribution of distances over the induced subgraph of each color in the cdBG that contains both unitigs (Fig. 1B). The mean of this distribution serves as an effective measure of their genomic proximity. To avoid calculation of MI for noninformative unitigs, we included options for filtering out unitigs based on their minor allele frequency (MAF) and the inferred genomic distance. The PAN-GWES method leverages the computational efficiencies of the SpydrPick algorithm (Pensar et al. 2019) to rapidly calculate the pairwise MI values of millions of unitigs pairs, allowing it to scale to large and diverse pangenomes such as those of *Escherichia coli*.

A critical component of the PAN-GWES pipeline is the initial choice of *k*-mer length. Longer *k*-mers are more specific and can capture fine-scale mutations. Conversely, shorter *k*-mers allow for greater sequence diversity and are better at capturing differences in gene content (Jaillard et al. 2018; Lees et al. 2019). Because obtaining a good balance between shorter and longer *k*-mers depends on the amount of diversity present in the target species, we allow the user to run the PAN-GWES pipeline repeatedly with different values of *k* (typically 31–201) to explore and compare the resulting LD patterns.

### Pangenome GWES analysis identifies novel links of coselection in *Streptococcus pneumoniae*

To verify that our graph-based distance estimates can identify signals of epistasis and coselection, we compared the results from our pangenome approach to the epistatic and coevolutionary hits identified by running the previously published SpydrPick algorithm on a MSA built from a collection of 3042 *Streptococcus pneumoniae* genomes (Chewapreecha et al. 2014). The genomes were sequenced from isolates collected in Maela, a refugee camp on the Thai-Myanmar border between 2007 and 2010. SpydrPick uses the distance between variants in an MSA, usually built by aligning sequencing reads to a reference genome, in this case the pneumococcal reference genome of a serotype 23F strain, ATCC 700669 (Croucher et al. 2009). This assumes that the distance between sites in the reference is a good proxy for the distance between sites in the full collection of genomes. The Maela collection of *S. pneumoniae* genomes was sequenced in 2010–2011 using short reads 75 bp in length. Consequently, the resulting assemblies are highly fragmented (average contig length of 33,191 bp and average N50 of 65,656 bp), which represents a challenge for our algorithm, as the resulting graph for each color may not always connect two unitigs. Despite this, there was a strong correlation between distances estimated using the graph-based approach to those based on alignment with the pneumococcal reference genome (Supplemental Fig. 6).

**Figure 3.**
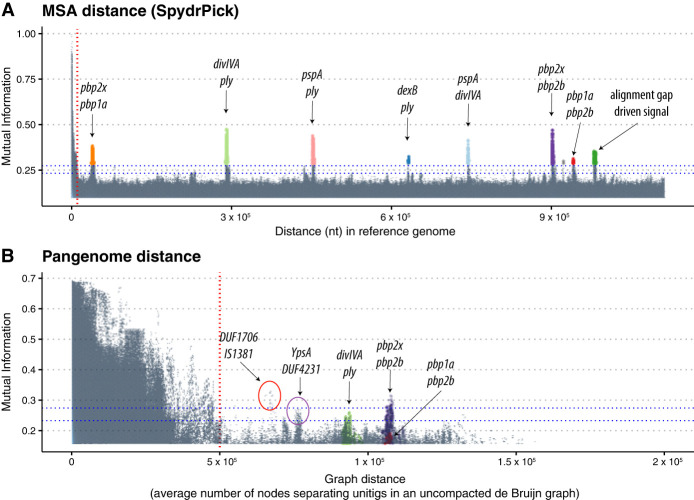
Manhattan plots indicating the strength of linkage disequilibrium (LD), using MI, versus the distance separating loci using a single reference and the SpydrPick algorithm (*A*) and the PAN-GWES algorithm (*B*). Separate colors are given to each link and are consistent between the plots. Unitig pairs with an average distance exceeding the standard deviation of their distances have been filtered out in the PAN-GWES results. Despite the highly fragmented nature of the data set reducing the sensitivity of the PAN-GWES approach, similar signals of coselection between the penicillin binding proteins were observed. The strongest link was observed in the PAN-GWES approach (red circle) between a DUF1706 domain–containing protein and the putative insertion sequence IS1381. In addition, the link between *ypsA* and a DUF4231-containing protein (purple circle) was obscured when relying on a single-reference genome, as the location of the insertion sequence varies considerably and DUF4231 is part of the accessory genome. The horizontal blue lines in the graph represent the “outlier” and “extreme outlier” thresholds inferred using Tukey's method (Methods). Points *above* these lines, which are separate from the large cluster driven by LD on the *left* side of both plots, can be interpreted as strong signals of coselection or epistasis. The green points in *A*, driven by gaps in alignment with the reference genome, would be represented as unitig-presence and -absence patterns in the graph-based approach. These signals did not appear in the PAN-GWES method, suggesting that they are likely caused by misalignment to the reference genome rather than by the actual presence or absence of sequence.

Figure 3 indicates that despite the difficulties caused by fragmentation, our pangenome distance-based approach is able to identify similar signals to the SpydrPick algorithm, albeit with some loss in sensitivity. Supplemental Figure 5 illustrates the LD-decay pattern based on specific choices of *k*-mer length and filtering criteria. Longer *k*-mers are more specific, resulting in larger distances within the pangenome graph. Shorter *k*-mers enable the identification of associations between more diverse features, including gene gain and loss. This is similar to the impact of *k*-mer length on bacterial GWAS, which has been described in detail in previous publications (Jaillard et al. 2018; Lees et al. 2019).

The strong coevolutionary signal between the penicillin-binding proteins (PBPs) *pbpX* and *pbp2B* was clearly identified using both algorithms (shown in purple). The PBP proteins are targets for beta-lactam antibiotics, and modifications in both of these proteins are the primary resistance mechanism for multiple classes of beta-lactams (Spratt 1994; Grebe and Hakenbeck 1996). Similarly, the pangenome-based algorithm identified strong links (colored in green) between SPN23F16620 (*divIVA*) and the gene cluster SPN23F19480–SPN23F19500, which is located directly upstream of the gene encoding the toxin and key virulence factor pneumolysin (*ply*/SPN23F19470). *divIVA* encodes a cell morphogenesis factor, and these coupling links have been hypothesized to be the consequence of virulence proteins interacting at the cell surface (Fleurie et al. 2014; Skwark et al. 2017).

In addition to rediscovering known associations, our pangenome graph approach detected a number of novel linked loci that were obscured when relying on only a single reference. This included associations between variants in a ligand-binding protein (*ypsA*), a Domain of Unknown Function 4231 (DUF4231) containing protein, present in the accessory genome. As DUF4231 is not present within the ATCC 700669 reference strain, it was not detectable using reference-based methods. Expression of DUF4231-containing proteins has been associated with both exposure to cigarette smoke and macrolides (Goad and Harris 2018; Manna et al. 2018). A DUF4231-containing protein is also part of the pneumolysis (*Ply*) operon, encoding a cytolytic pore-forming toxin, and a primary virulence factor for this bacterium (Stevens et al. 2022). Within-host variation in *ypsA* has been linked with penicillin treatment in the same cohort using a unitig-based GWAS (Tonkin-Hill et al. 2022). This variation was hypothesized to allow pneumococci to reduce their metabolism and cell division, allowing the population to persist in periods of stress when the antibiotic was present. The association between these two loci could be because of their involvement in the same stress-response pathways.

The association with the highest MI value for distant unitigs identified using our pangenome approach (Fig. 3, red circle) was found between SPN23F08610 (*dfsB*), a DUF1706 domain-containing protein, and the putative insertion sequence IS1381 (SPN23F08630), found in the accessory genome. dfsB is widely distributed across bacterial species and has been associated with inducing cell death in nearby colonies of bacteria (Taylor et al. 2016). The average distance between unitigs from these genes in the de Bruijn graph was 67,000 bp. However, the multicopy IS-element is highly mobile and has been observed to insert close (<2000 bp) to *dfsB* in several assemblies, including ATCC-700669 (Supplemental Fig. 4). The link with an insertion sequence could be associated with phenotypic switching caused by the movement of mobile elements, or alternatively, it could be an artifact introduced by the highly fragmented assemblies in the Maela collection. To further validate the identified coevolutionary signal, it is important to apply the PAN-GWES algorithm to enhanced assemblies, potentially using long-read technology.

### Accurate long-read assemblies reveal new evidence of coselection and epistasis in *E. faecalis*

**Figure 4.**
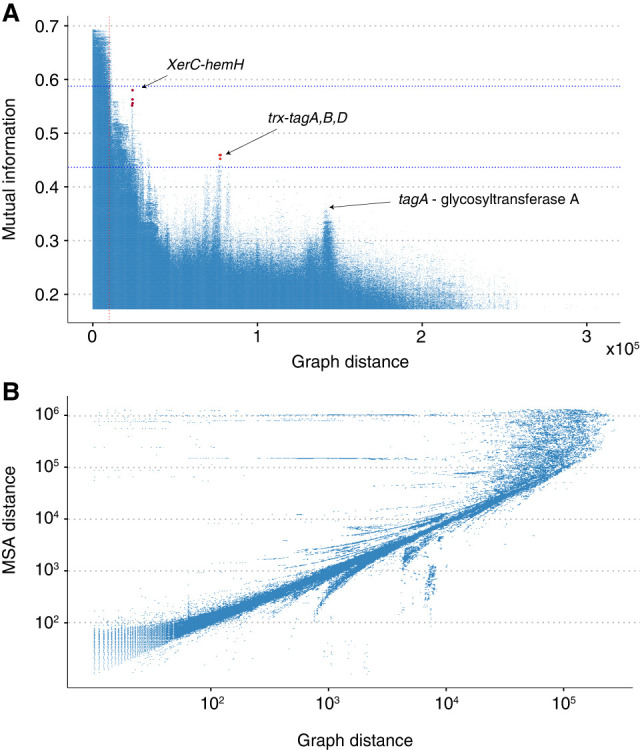
Analysis of long-read *E. faecalis* assemblies. (*A*) A Manhattan plot indicating the strength of LD measured using MI versus the genomic distance in the graph. Unitig pairs with an average distance exceeding the standard deviation of their distances have been filtered out. Strong links of coselection were identified between an intergenic region adjacent to the *trxB* gene and the three genes *tag*A, -B, and -D in addition to a link between a site-specific *XerC* family tyrosine recombinase and an intergenic region between a tRNA gene and a ferrochelatase-encoding hemH. The vertical red line indicates the distance threshold below which it is difficult to distinguish outlier peaks from the background LD distribution. Pairs with MI values significantly above the background at a given distance are likely to be impacted by either epistasis or coselection. (*B*) Pairwise distances between loci within a single genome and those found using the PAN-GWES graph-based approach. The correlation, particularly over shorter distances, indicates that the PAN-GWES algorithm can accurately distinguish LD driven by proximity within the genome from LD indicative of coselection or epistasis. The horizontal lines are caused by *k*-mers associated with mobile elements that appear in different locations within each genome but are fixed within the single reference.

As the graph-based genomic distance approximation is expected to be most accurate with complete genome assemblies, in which longer pangenome distances can be accurately inferred, we tested our method on a large and representatively sampled *E. faecalis* data set consisting of Illumina and Oxford Nanopore Technologies hybrid assemblies (Supplemental Table 2; Pöntinen et al. 2021). In total, we considered 332 complete circular chromosomes, combined with 43 unpublished near-complete chromosomes from the same population, leading to a total of 375 genomes. The stability of the LD signals as a function of approximated genomic distance demonstrates the attractive features of long-read-based assemblies as an input to a pangenomic GWES analysis (Fig. 4A). Moreover, a comparison of chromosome distances calculated using a single genome and the PAN-GWES method was highly correlated, indicating the algorithm provides an informative measure of distance (Fig. 4B).

Screening of the top hits within the *E. faecalis* collection resulted in 66 unitig pairs, further filtered to 29 unique pairs when considering individual genes and their intergenic regions (Supplemental Table 1). Of these, intergenic regions were overrepresented, as 65.5% (19/29) of hit pairs exhibited an intergenic region in at least one of the pairs of unitigs. To assess whether any signals were associated with the clinical setting, the presence of top hits was compared between isolates from hospital and nonhospital sources, with the overrepresentation of intergenic hits also reflected in the hospital-associated isolates. Five unique hits were more prevalent in *E. faecalis* isolates from hospitalized patients compared with other sources (Supplemental Table 1), four of which included the intergenic region between a hypothetical protein and a thioredoxin reductase–encoding *trxB* (chi-square test, *P* < 0.05) (Supplemental Table 1). *TrxB* is a conserved detoxifying enzyme and part of the thioredoxin complex, globally involved in the oxidative stress response (Zeller and Klug 2006; Kajfasz et al. 2012). In addition to another hypothetical protein, the *trxB* intergenic hit was separately linked to three genes (*tagA*, *tagB*, and *tagD*) from the cell wall teichoic acid synthesis pathway, of which intact tagB has been indicated with a role in evading the complement system activation in a host by modification of peptidoglycan (Geiss-Liebisch et al. 2012). Oxidative stress, both in the environment and during intracellular host infection, and the hurdles of the host immune system are both conditions that *E. faecalis* would frequently face in the hospital setting, potentially explaining the multiple links between the two functions. Another hit enriched in hospital isolates was identified between a site-specific *XerC* family tyrosine recombinase and an intergenic region between a tRNA gene and a ferrochelatase-encoding *hemH*, which adds iron to heme. This link could be explained by the connection of the integrase gene of a phage to its integration site, perhaps more so than directly to the ferrochelatase. As intergenic regions often harbor regulatory machinery of the nearby genes, their overrepresentation within the top hits demonstrate the potential of our phenotype-free approach to uncover regulatory signals that can be important in pathogenesis and that usually would be detectable from population transcriptomic data only (Kachroo et al. 2019). Although *trxB* is a core gene in *E. faecalis*, the remaining hits are part of the accessory genome and can only be identified using previous methods if a reference genome containing these loci is used. In this case, we used a close reference, E00113 (ERR4406486), as it contained most of the unitig hits, allowing for a comparison of distance estimates. This reference included the *hemH* and *XerC* loci, allowing them to be visualized in the equivalent reference-based Manhattan plot (Supplemental Fig. 7). However, these loci were located more closely together in the reference genome than in the broader population, and as a result, they would likely have been filtered out in a classic SpydrPick analysis.

## Discussion

The decreasing cost and improved scalability of both short- and long-read sequencing are continuing to rapidly increase the availability of high-quality population genomic data for many bacterial species, in particular for those with relevance to public health. Currently there is untapped potential in using these data to study bacterial evolution and adaptation. GWES is a recently emerged tool for uncovering drivers of change in genomes through LD pattern analysis without assuming availability of phenotypic data from population-wide screening. However, currently these approaches are limited to consider only the core genome. To fill this gap, we introduced a PAN-GWES approach that allows for a more holistic view over the population patterns of genomic covariation.

We identified strong associations between known antibiotic-resistance genes in *S. pneumoniae* in addition to links enriched in hospital-derived *E. faecalis* isolates, which demonstrate the potential of our approach to identify genes and regions within the genome that could be the target of future studies or interventions aimed at reducing the burden of infectious disease. A key innovation in our method is the use of colored and compacted de Bruijn graphs to obtain a computationally scalable measure of the genomic distance between loci within a species pangenome.

Although highly fragmented assemblies represent a challenge for our algorithm, we demonstrate that although it can lead to a reduction in sensitivity over reference-based approaches, we are nevertheless able to identify signals of epistasis and coselection that would be missed by previous approaches. Notably, the “missing” signal identified in our analysis of the pneumococcal pangenome included genes previously associated with antimicrobial treatment. This further demonstrates the ability of our method to detect clinically important associations without requiring phenotypic metadata. When dealing with highly fragmented data sets, we recommend the use of both reference-based methods, such as SpydrPick, and PAN-GWES to take advantage of the strengths of both tools. As advancements in long-read technologies continue to deliver increased precision and affordability, we anticipate that genome assemblies will shift toward completeness or near-completeness as the standard. Consequently, the efficacy of our algorithm is likely to improve, providing more accurate inferences on contemporary genome data sets, as exemplified in our *E. faecalis* analysis.

## Methods

### de Bruijn graph construction

Let *S* = (*S*_1_, …, *S*_*N*_) be a set of *N* assembled DNA sequence strings over the DNA alphabet Σ = {*A*, *C*, *G*, *T*}. A substring of length *k* that is contained within a sequence in *S* is called a *k*-mer. Given a *k-*mer *s*, let $\bar{s}$ denote the reverse complement of *s*, which is formed by reversing the string and interchanging A and T and interchanging C and G. We denote by $\hat{s}$ the lexicographically smaller string between *s* and $\bar{s}$, which is called the canonical *k*-mer. Using canonical *k-*mers allows us to account for each location in a genome once while accounting for both reverse complementary sequences. For a *k*-mer *s*, pre(*s*, *l*), and suf(*s*, *l*), denote the prefix and the suffix of length *l* of *s*, respectively.

Given a set of strings *S* and an odd integer *k* > 0, we define the de Bruijn graph as a bidirected multigraph *G*_*S*,*k*_ = (*V*, *E*), where the set of vertices *V* is exactly the set of canonical *k*-mers contained in the sequences in *S*. Two vertices *v* and *w* in *V* are connected by an edge *e* if and only if there exists a (*k* + 1)-mer *z* in *S* such that $\mathrm{pre}(\hat{z},k)=v$ and $\mathrm{suf}(\hat{z},k)=w$. The choice of an odd *k*-mer length ensures that a *k*-mer can never be its own reverse complement, which would cause ambiguity in the graph. A sequence of distinct vertices is called a path if every two adjacent vertices in the path are connected by an edge in *G*. The path *p* = (*v*_1_, …, *v*_*m*_) is called nonbranching if (1) each internal vertex *v*_*i*_, *i* = 2, …, *m* − 1, has exactly two edges that connect to different sides of *v*_*i*_ and (2) the first and last vertices *v*_1_ and *v*_*m*_ have exactly one edge on the side which connects to *v*_2_ and *v*_*m*−1_, respectively. A nonbranching path is said to be maximal if the path cannot be extended by a vertex on either side without branching. For convenience, maximal nonbranching paths are reduced to a single vertex, referred to as a “unitig,” leading to a cdBG.

### Colored de Bruijn graph

We can extend the cdBG to include colors representing which *k*-mer is present in which genome. A colored de Bruijn graph is a graph *G* = (*V*, *E*, *C*) in which (*V*, *E*) is a dBG and *C* is a set of colors such that each vertex *v* ∈ *V* maps to a subset of *C*. A path through this graph is called color-coherent if all its vertices share the same color set. We define the colored cdBG as a bidirected multigraph *G*_*S*,*k*_ = (*V*, *E*, *C*), which is constructed by collapsing all of its maximal color-coherent nonbranching paths into single vertices, which are called unitigs.

### Genomic distance calculation

In *G*_*S*,*k*_, the length of a path is simply the number of edges in the path. However, a path *p* = (*v*_1_, …, *v*_*m*_) in the cdBG obtained from *G*_*S*,*k*_ consists of unitigs that in themselves are paths in *G*_*S*,*k*_. Therefore, to measure the length of *p*, special attention must be given to the actual number of edges traversed with respect to the uncompacted graph. We define the shortest distance between two unitigs, *p* and *q*, in *V* to be the length of the path starting from *p* and ending at *q*, traversing via edges in the uncompacted graph, with minimal length. The path is calculated using a parallel version of Dijkstra's algorithm (Dijkstra 1959). To define the distance between unitigs in a colored cdBG, we calculate the distance between *p* and *q* for each subgraph *H* ∈ *G*, where *H* includes only those vertices in a single color in *C*. The average distance over all colors is then taken.

### MI and the SpydrPick algorithm

PAN-GWES uses the SpydrPick algorithm to calculate MI (Pensar et al. 2019). The MI between two random variables quantifies the level of dependence between them. We consider two discrete random variables, *X* and *Y*, which represent the presence (one) and absence (zero) of two unitigs, respectively. The MI between *X* and *Y* can then be written as

$$MI(X,Y)=\underset{x\in \{0,1\}}{\sum }\underset{y\in \{0,1\}}{\sum }p(x,y)\log\left(\frac{\;p(x,y)}{\;p(x)p(y)}\right),$$

where *p*(*x*, *y*) is the joint probability distribution function of *X* and *Y*, indicating the probability of *X* and *Y* simultaneously taking specific values. $p(x)=\sum_{y\in \{0,1\}}p(x,y)$ and $p(y)=\sum_{x\in \{0,1\}}p(x,y)$ are the marginal probability distributions of *X* and *Y*, respectively, indicating the probabilities of observing specific values independently in each variable.

To estimate these distributions, let *n*(*x*, *y*) be the count of each combination of presence and absence of a given pair of unitigs within the data set. To avoid issues with zero counts we calculate

$$\hat{\;p}(x,y)=\frac{n(x,y)+0.5}{n+r_{X}r_{Y}0.5},$$

where *n* is the total number of samples, and *r*_*X*_ = |*val*(*X*)| and *r*_*Y*_ = |*val*(*Y*)| are the observation counts of either the presence or absence of each unitig, respectively.

To control for population structure and the dependence structure between samples within a data set, the contribution of each genome is reweighted by how different it is from other genomes within the data set. The number of sequences, *m*_*i*_ with a mean site Hamming distance less than a threshold ε is calculated for each genome *G*_*i*_. A genome-specific weighting is then calculated as

$$w_{i}=\frac{1}{m_{i}}.$$

The default threshold used by SpydrPick and PAN-GWES is ε = 0.1. The effective count *n*_*eff*_(*x*, *y*) is then calculated as

$$n_{eff}(x,y)=\underset{i}{\sum }\frac{1}{m_{i}}1_{X=x}1_{Y=y},$$

which can be substituted into the equation for $\hat{\;p}(x,y)$.

For large data sets, storing the MI values for a very large number of possible unitig pairs can be prohibitive. As only the largest MI values are of interest, the SpydrPick algorithm subsamples pairs to retain only the top fraction of MI values. A suitable MI threshold for retention is calculated by randomly selecting a subset of unitig pairs for which the MI values are calculated. The empirical cumulative distribution function is then used to estimate an appropriate saving threshold that aligns with a user-specified top fraction (default = 25%).

The ARACNE algorithm can optionally be used to select only the most promising links. It examines each triplet of unitigs and retains the two links with highest MI. This helps reduce the impact of hitchhiking mutations, which are driven by LD, which can lead to an inflation in the number of reported links. An extended description of this algorithm, including an illustrative figure, is provided in the original description of the SpydrPick algorithm (Pensar et al. 2019). Finally, the most promising unitig pairs are selected using a Tukey outlier analysis (Tukey 1977). This involves calculating the upper (*Q*_1_) and lower (*Q*_3_) quarterlies for all MI values that pass the initial filters. Following Tukey's criterion, unitig pairs with an MI value greater than *Q*_3_ + 1.5 × (*Q*_3_ − *Q*_1_) are classified as outliers, and unitig pairs with an MI value larger than *Q*_3_ + 3 × (*Q*_3_ − *Q*_1_) are classified as extreme outliers.

### Filtering

Depending on the quality of the genome under consideration, it can be important to filter potential unitig pairs to focus only on the most promising ones. In addition to choosing a suitable *k*-mer length, we suggest several main filters, including filtering based on the frequency of a unitig pair within the data set, the average distance in the graph between pairs, and the variability in this distance. The impact of these filters on the pneumococcal data set is shown in Supplemental Figure 1.

We recommend filtering out links between unitigs that are located too close together within the de Bruijn graph. A suitable distance threshold can be inferred by plotting the number of unitig pairs versus their distance in the graph (Supplemental Fig. 2). The elbow point or the distance at which this distribution levels out provides a reasonable threshold for excluding pairs that are likely to be more strongly impacted by LD. Pairs with at least 5 × 10^4^ and 1 × 10^4^ nodes separating them in the uncompacted de Bruijn graph were considered in the analysis of the *S. pneumoniae* and *E. faecalis* data sets, respectively. As each unitig pair may be at different distances in each genome, the variance in these distances can also be used to filter out potential noise. We generally recommend considering pairs in which the ratio of the average distance to the standard deviation of their distance in each subgraph (represented by an individual color in the full de Bruijn graph) is at least one (Supplemental Fig. 3). Increasing this threshold will focus on those unitig pairs that are consistently located at a sufficient distance in the graph, thereby reducing the impacts of LD.

Ultimately, the choice of filtering thresholds in PAN-GWES depends on the data set characteristics and specific analysis goals. When experimental validation resources are limited, researchers may opt for stringent filters to prioritize the most statistically significant associations. Conversely, studies aiming to generate a comprehensive set of potential interacting loci for comparison with existing literature might prefer a more relaxed filtering approach. It is crucial to remember that although PAN-GWES identifies associations after controlling for population structure, true biological interactions require independent experimental validation. Generally, the most compelling candidates are those that show the greatest deviation in MI from other pairs of unitigs at similar average genetic distances.

### Implementation

The PAN-GWES program is an open-source program implemented in C++ that handles the efficient construction of the color-induced de Bruijn subgraphs and the following shortest path distance calculations. It supports parallel execution and further improves run time by smart arrangement of the graph search jobs. The program also incorporates a convenient parser for files formatted in Graphical Fragment Assembly 1.0 (GFA1) in order to construct all the necessary files used in the full pipeline.

The full PAN-GWES pipeline consists of four stages. First, Cuttlefish is utilized to construct the colored cdBG in GFA1 format, which is then parsed by PAN-GWES to prepare various graph data files with color information and a unitig frequency file to be used as an input file for SpydrPick (Pensar et al. 2019; Khan and Patro 2021). Next, the list of top candidate unitig pairs is calculated with the SpydrPick algorithm. Finally, the PAN-GWES program is used to calculate the distances between unitigs in the subgraph containing the nodes present in a single genome within the full colored de Bruijn.

### Sequencing, assembly, and variant calling

All the circular chromosome sequences of the fully contiguous hybrid assemblies (n = 332) were included from the previously collated *E. faecalis* data set, obtained from the NCBI BioProject database (https://www.ncbi.nlm.nih.gov/bioproject/) under accession number PRJEB28327 (Pöntinen et al. 2021). The collection was supplemented with 43 new, partially contiguous, *E. faecalis* hybrid assemblies, constructed from Illumina short-read and Oxford Nanopore Technologies (ONT) long-read sequences using a hybrid assembly pipeline (https://github.com/arredondo23/hybrid_assembly_slurm) with Unicycler v.0.4.7 (Wick et al. 2017). The newly introduced isolates were delineated into clusters using PopPUNK (Lees et al. 2019) v.1.1.5 with the ‐‐assign-query mode against the previously curated *E. faecalis* database and clustering scheme (Pöntinen et al. 2021). The unique PAN-GWES top hits were annotated using annotate_hits_pyseer of the pyseer tool v.1.3.9 (Lees et al. 2018) against selected hybrid assemblies from the collection as references to cover all of the top hits. Unitig-caller v.1.3.0 (Holley and Melsted 2020; Lees et al. 2020) with ‐‐simple mode was used for creating presence/absence matrix of the top hit pairs across the *E. faecalis* collection. Annotated hits were manually curated, and hit pairs allocated with the same annotations were merged. To test the significance of differences in proportions of each PAN-GWES top hit pair between hospital- and non-hospital-associated *E. faecalis* isolates, a two-sided Fisher's exact test was used when there were any counts less than five and Pearson's chi-squared test was used with higher counts than five. Both tests were performed with a significance threshold of *P* < 0.05.

The original *S. pneumoniae* genome assemblies from Chewapreecha et al. (2014) were used as input to PAN-GWES. The raw pneumococcal sequencing reads are available from the NCBI Sequencing Read Archive (SRA; https://www.ncbi.nlm.nih.gov/sra) under the original study accession numbers ERP000435, ERP000483, ERP000485, ERP000487, ERP000598, and ERP000599.

### Software availability

The PAN-GWES program is available under a MIT license from GitHub (https://github.com/jurikuronen/PANGWES) and as Supplemental Code. Further examples and conda installation instructions are available at GitHub (https://github.com/Sudaraka88/PAN-GWES).

## Data access

The Nanopore sequencing data generated in this study have been submitted to the NCBI BioProject database (https://www.ncbi.nlm.nih.gov/bioproject/) under accession number PRJEB40976.

## Supplementary Material

Supplement 1

Supplement 2

Supplement 3

Supplement 4

Supplement 5

Supplement 6

Supplement 7

Supplement 8

Supplement 9

Supplement 10
