# Supplementary material for: Pangenome-spanning epistasis and coselection analysis via de Bruijn graphs
Source: Genome Res. 2024 Jul;34(7):1081–8. doi: 10.1101/gr.278485.123 (PMC11368177; doi:10.1101/gr.278485.123)
Supplement: Supplement 1 [file Supplemental_Fig_S1.pdf]

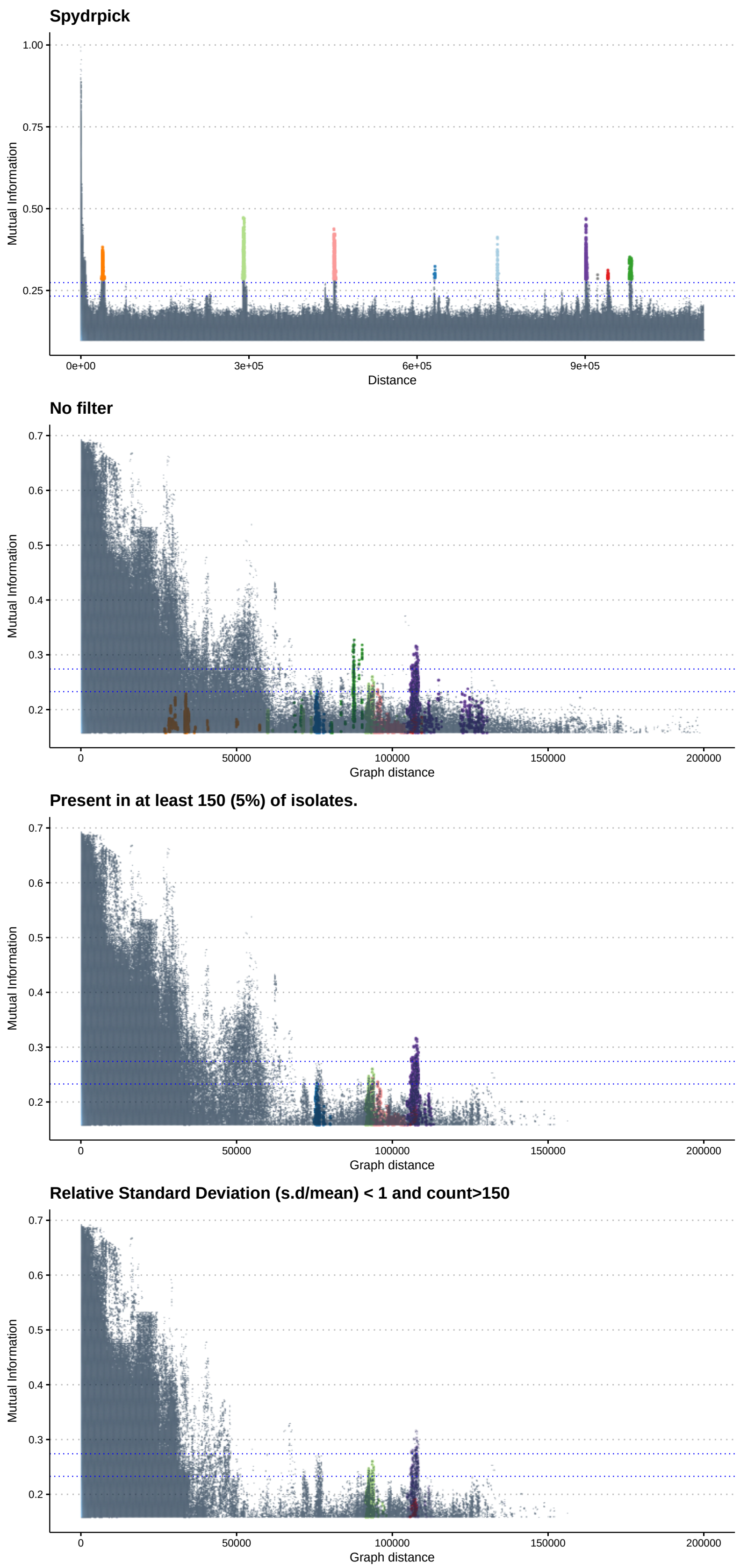

Supplementary Figure 1. Manhattan plots indicating the distribution of MI values by distance with different filters in the PAN-GWES pipeline applied to the *S. pneumoniae* dataset. The top facet indicates the results of the Spydrpick algorithm that relies on a reference genome alignment.
