# Supplementary material for: Pangenome-spanning epistasis and coselection analysis via de Bruijn graphs
Source: Genome Res. 2024 Jul;34(7):1081–8. doi: 10.1101/gr.278485.123 (PMC11368177; doi:10.1101/gr.278485.123)
Supplement: Supplement 2 [file Supplemental_Fig_S2.pdf]

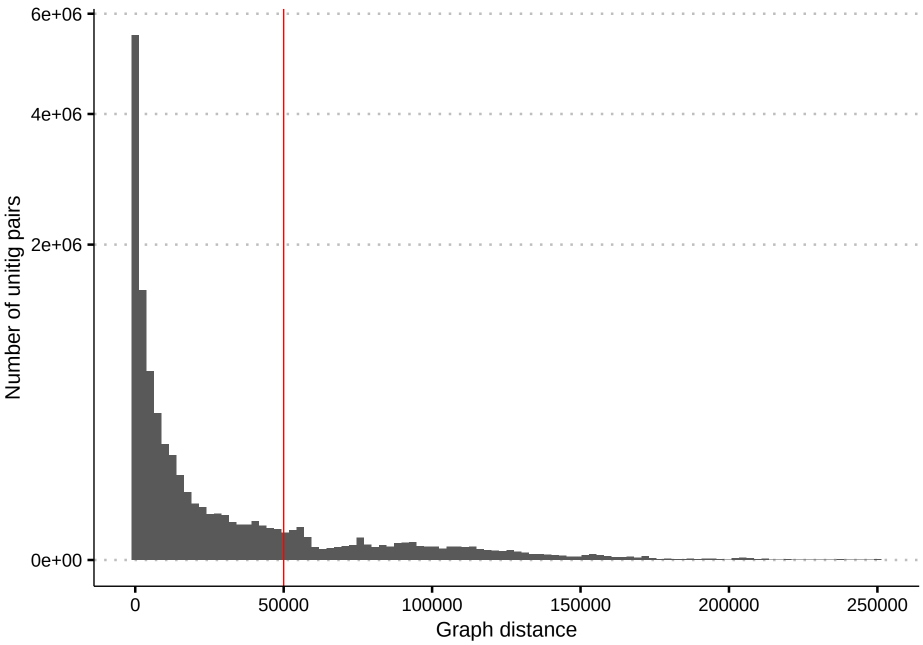

Supplementary Figure 2. A histogram indicating the number of unitig pairs versus the average distance across each colour in the coloured de-Bruijn graph for the pneumococcal dataset. Only pairs above 50,000bp were considered in the final analysis.
