# Supplementary material for: Pangenome-spanning epistasis and coselection analysis via de Bruijn graphs
Source: Genome Res. 2024 Jul;34(7):1081–8. doi: 10.1101/gr.278485.123 (PMC11368177; doi:10.1101/gr.278485.123)
Supplement: Supplement 3 [file Supplemental_Fig_S3.pdf]

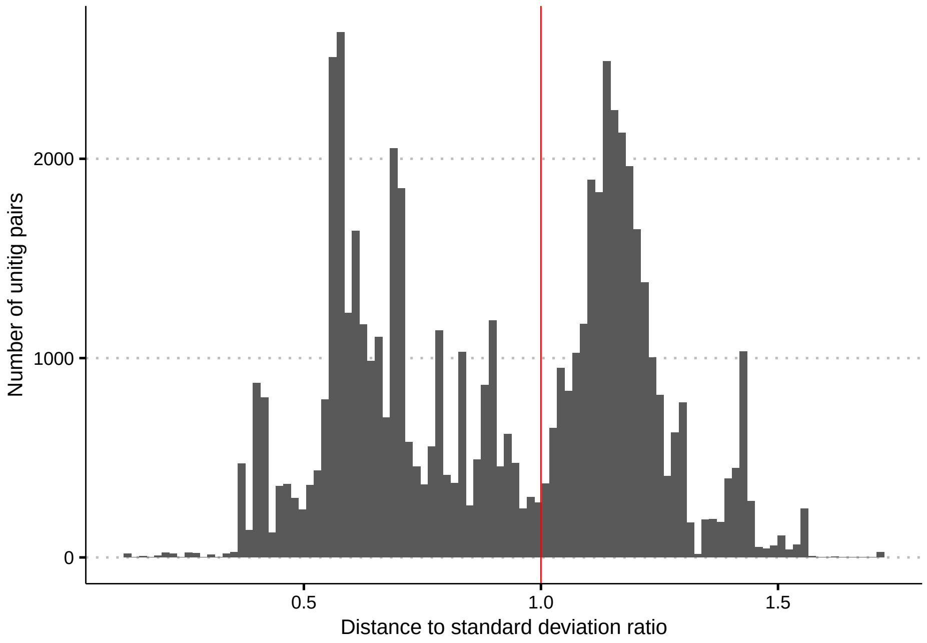

Supplementary Figure 3. The number of unitig pairs versus the standard deviation of their distance in each subgraph represented by an individual colour in the full coloured de Bruijn graph.
