# Supplementary material for: Pangenome-spanning epistasis and coselection analysis via de Bruijn graphs
Source: Genome Res. 2024 Jul;34(7):1081–8. doi: 10.1101/gr.278485.123 (PMC11368177; doi:10.1101/gr.278485.123)
Supplement: Supplement 4 [file Supplemental_Fig_S4.pdf]

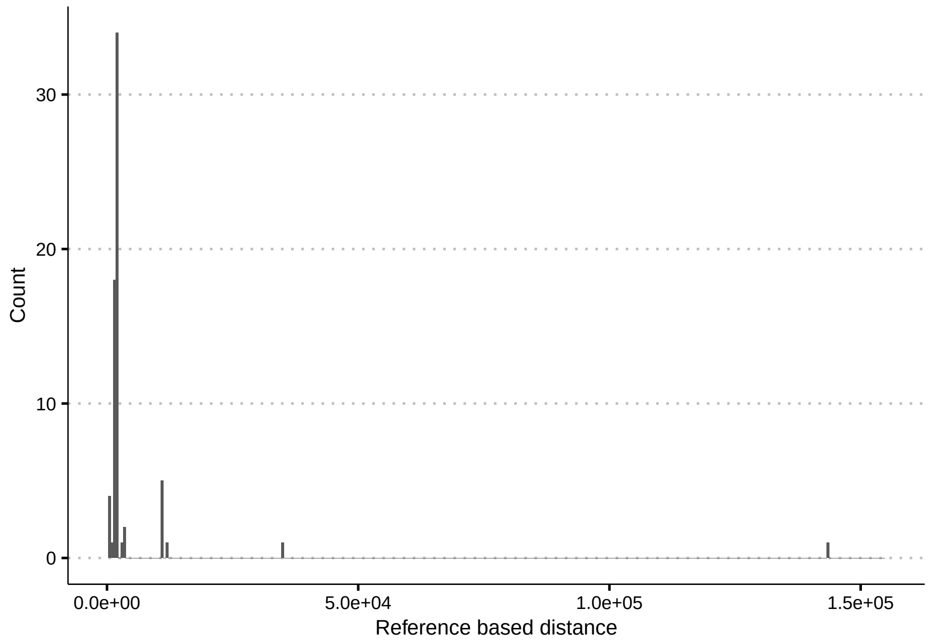

Supplementary Figure 4. A histogram of the distances between two unitigs linked to SPN23F08610 (dfsB), a DUF1706 domain-containing protein and the putative insertion sequence IS1381 (SPN23F08630) when they appeared on the same contig of an assembly. The cluster of short distances indicates that this link may be impacted by the highly fragmented assemblies in this dataset and highlights the importance of subsequent laboratory experiments to confirm identified associations.
