# Supplementary material for: Pangenome-spanning epistasis and coselection analysis via de Bruijn graphs
Source: Genome Res. 2024 Jul;34(7):1081–8. doi: 10.1101/gr.278485.123 (PMC11368177; doi:10.1101/gr.278485.123)
Supplement: Supplement 5 [file Supplemental_Fig_S5.pdf]

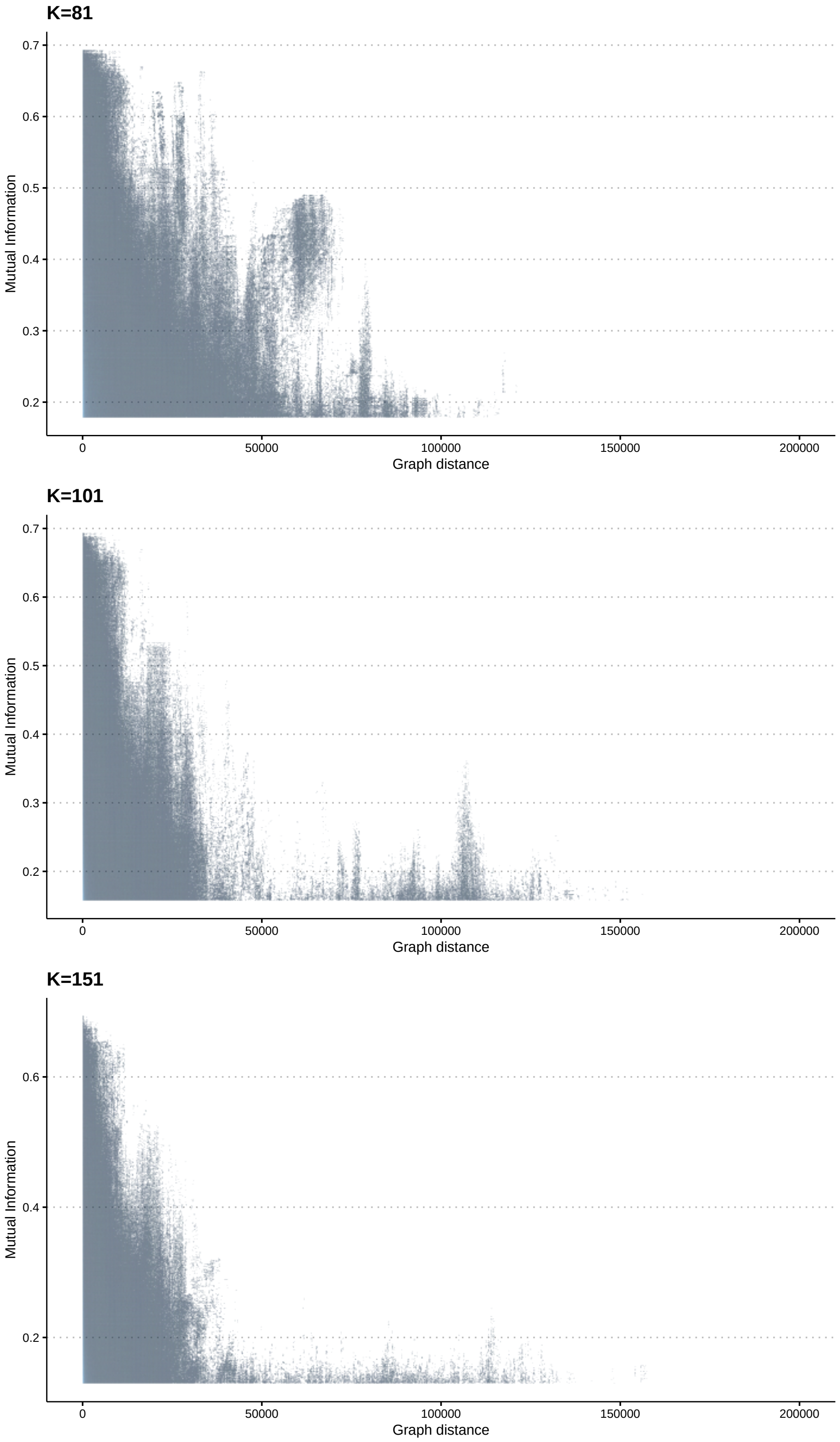

Supplementary Figure 5. Manhattan plots indicating the distribution of MI values by distance with different *k-mer* lengths in the PAN-GWES pipeline applied to the *S. pneumoniae* dataset.
