# Supplementary material for: Pangenome-spanning epistasis and coselection analysis via de Bruijn graphs
Source: Genome Res. 2024 Jul;34(7):1081–8. doi: 10.1101/gr.278485.123 (PMC11368177; doi:10.1101/gr.278485.123)
Supplement: Supplement 6 [file Supplemental_Fig_S6.pdf]

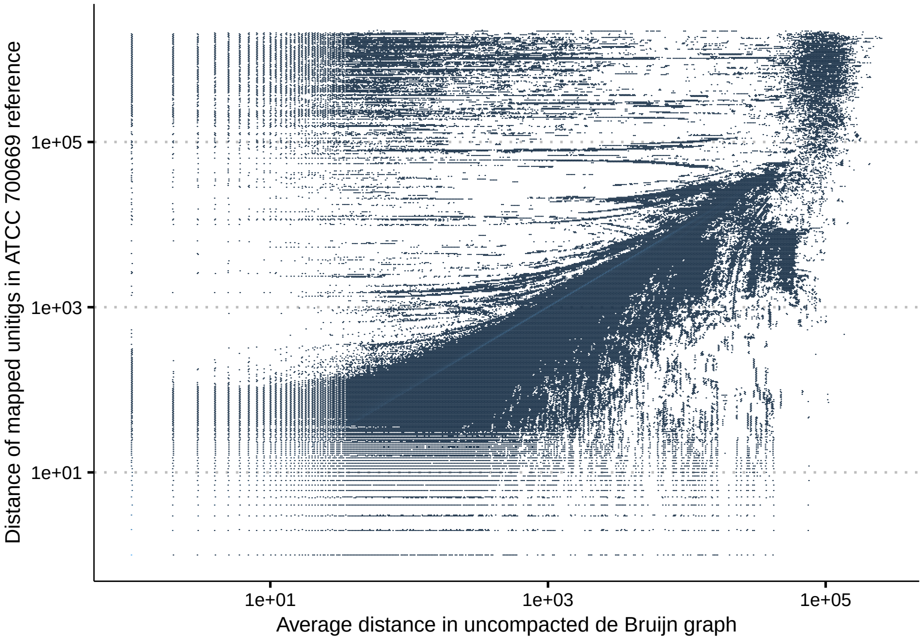


**Supplementary Figure 6.** The graphical distance in terms of the average number of nodes along the uncompacted de Bruijn graph versus the distance between unitigs mapped to the pneumococcal reference genome.
