# Supplementary material for: Pangenome-spanning epistasis and coselection analysis via de Bruijn graphs
Source: Genome Res. 2024 Jul;34(7):1081–8. doi: 10.1101/gr.278485.123 (PMC11368177; doi:10.1101/gr.278485.123)
Supplement: Supplement 7 [file Supplemental_Fig_S7.pdf]

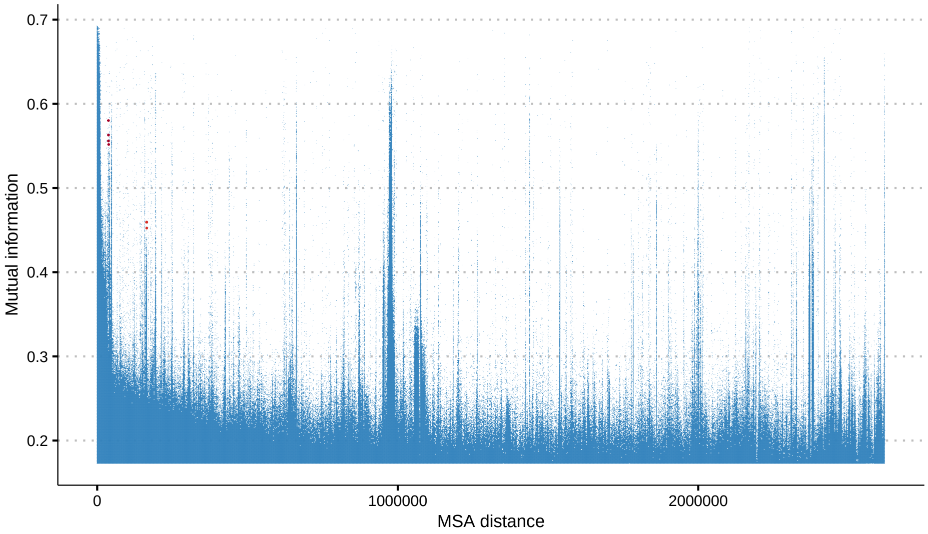

Supplementary Figure 7. The Manhattan plot produced from running Spydrpick on the *E. faecalis* data using a reference based distance. An internal reference, E00113 (ERR4406486) was selected as it harboured most of the unitig hits and had a hybrid complete circular genome assembly.
