# Supplementary figures and images for: Pangenome-spanning epistasis and coselection analysis via de Bruijn graphs
Source: Genome Res. 2024 Jul;34(7):1081–8. doi: 10.1101/gr.278485.123 (PMC11368177; doi:10.1101/gr.278485.123)

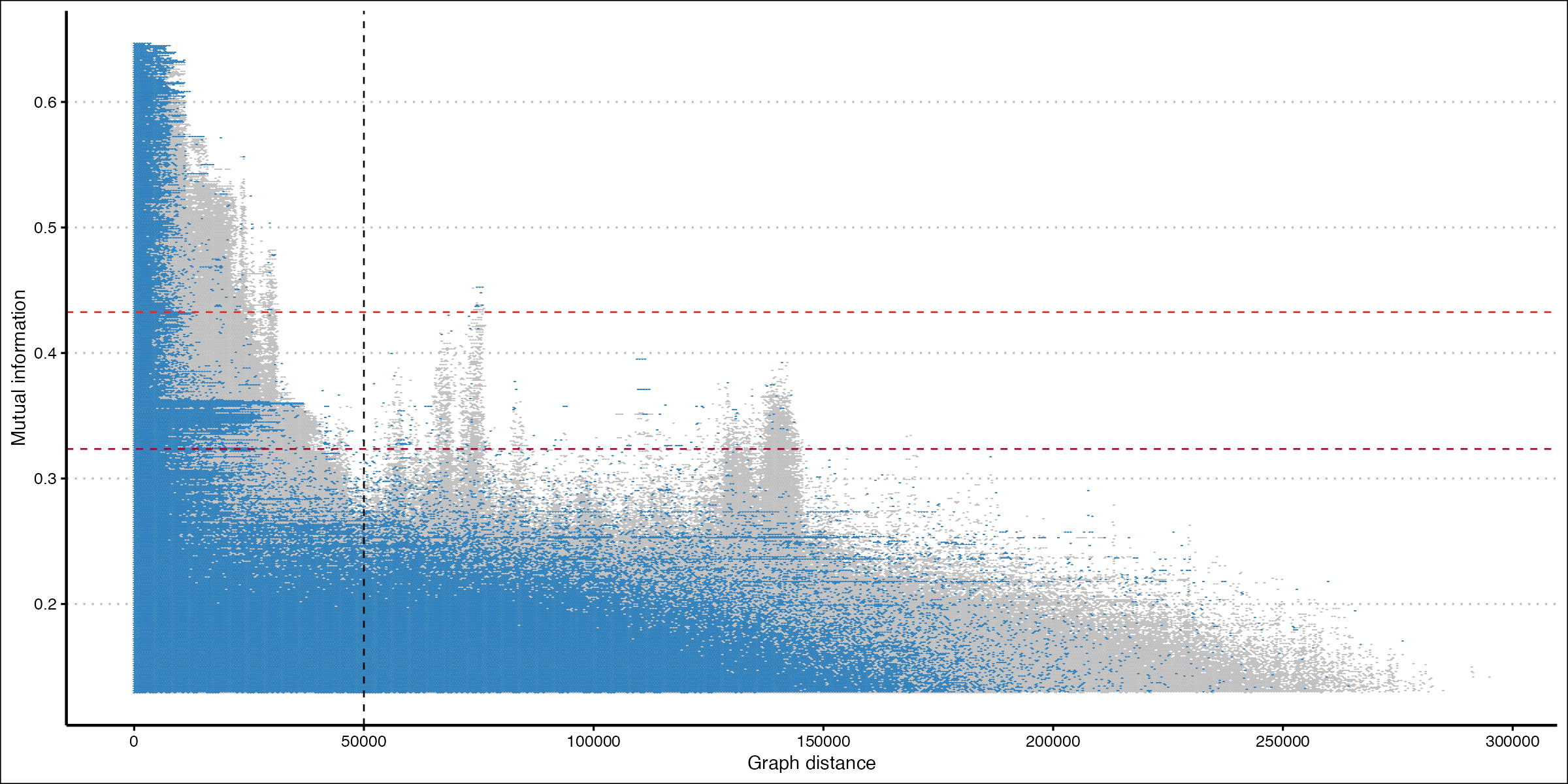

Supplement: Supplement 10 [file Supplemental_Code.zip › PAN-GWES-main/sw/pangwes/scripts/pg_fast.png]

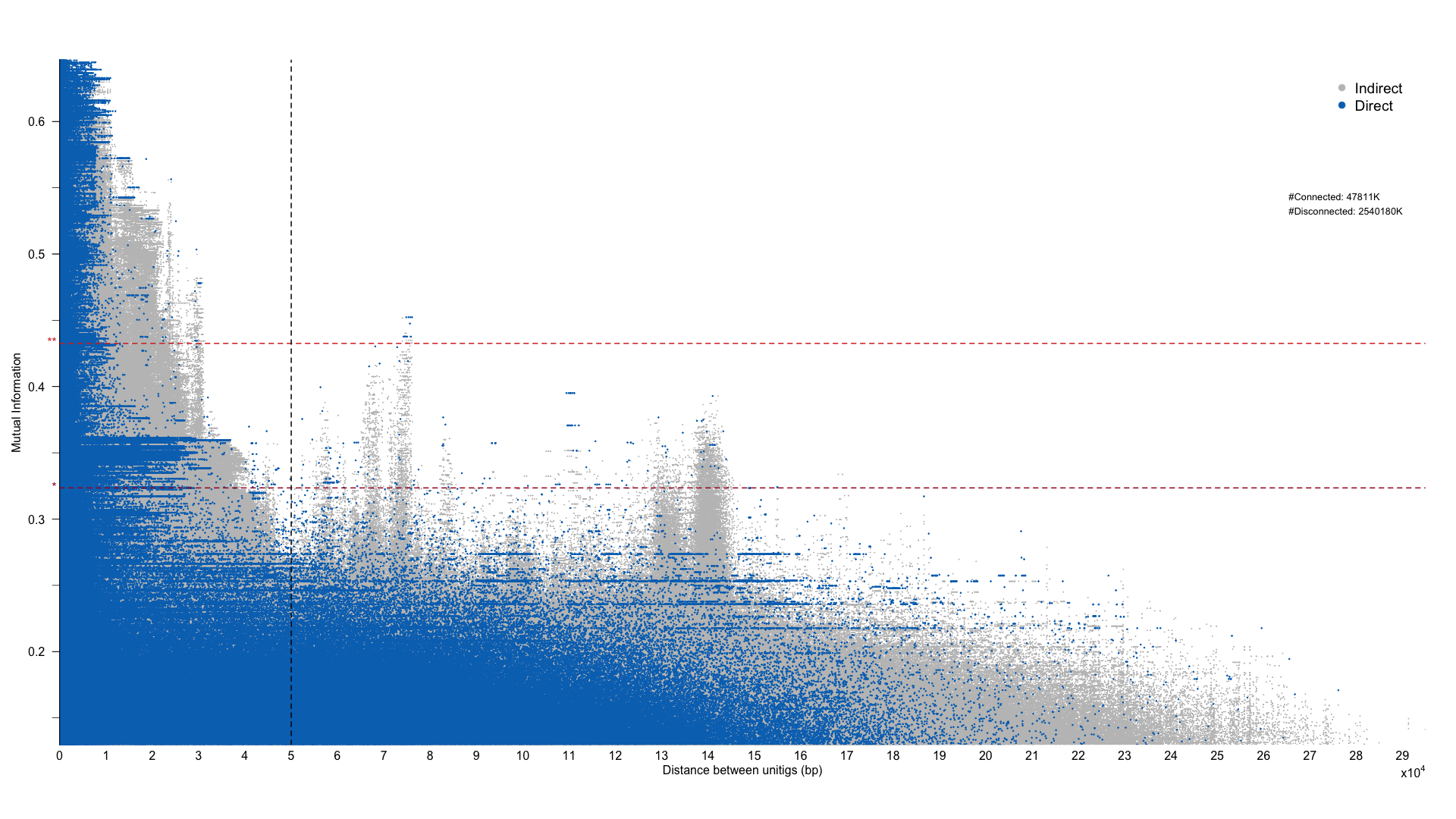

Supplement: Supplement 10 [file Supplemental_Code.zip › PAN-GWES-main/sw/pangwes/scripts/pg_slow.png]
